# Validation of the prevalence to incidence conversion method for healthcare associated infections in long-term care facilities

**DOI:** 10.1371/journal.pone.0300794

**Published:** 2024-03-21

**Authors:** Costanza Vicentini, Enrico Ricchizzi, Antonino Russotto, Stefano Bazzolo, Catia Bedosti, Valentina Blengini, Dario Ceccarelli, Elisa Fabbri, Dario Gamba, Anna Maddaleno, Edoardo Rolfini, Margherita Tancredi, Carla Maria Zotti

**Affiliations:** 1 Department of Public Health and Paediatrics, University of Turin, Torino, Italy; 2 Settore Innovazione nei Servizi Sanitari e Sociali, Direzione Generale cura della Persona, Salute e Welfare, Regione Emilia-Romagna, Bologna, Italy; 3 Department of Environment, Land and Infrastructure Engineering (DIATI), Politecnico of Turin, Torino, Italy; 4 Nucleo Operativo Rischio Infettivo—Governo Clinico—AUSL di Imola, Bologna, Italy; 5 RSA “Sant’Anna”, Pianezza, Torino, Italy; 6 University of Turin, Torino, Italy; 7 RSA “Aldo Maritano”, Sangano, Torino, Italy; Gabriele d’Annunzio University of Chieti and Pescara: Universita degli Studi Gabriele d’Annunzio Chieti Pescara, ITALY

## Abstract

**Introduction:**

Residents of long-term care facilities (LTCFs) are a population at high risk of developing severe healthcare associated infections (HAIs). In the assessment of HAIs in acute-care hospitals, selection bias can occur due to cases being over-represented: patients developing HAIs usually have longer lengths of stays compared to controls, and therefore have an increased probability of being sampled in PPS, leading to an overestimation of HAI prevalence. Our hypothesis was that in LTCFs, the opposite may occur: residents developing HAIs either may have a greater chance of being transferred to acute-care facilities or of dying, and therefore could be under-represented in PPS, leading to an underestimation of HAI prevalence. Our aim was to test this hypothesis by comparing HAI rates obtained through longitudinal and cross-sectional studies.

**Methods:**

Results from two studies conducted simultaneously in four LTCFs in Italy were compared: a longitudinal study promoted by the European Centre for Disease Prevention and Control (ECDC, HALT4 longitudinal study, H4LS), and a PPS. Prevalence was estimated from the PPS and converted into incidence per year using an adapted version of the Rhame and Sudderth formula proposed by the ECDC. Differences between incidence rates calculated from the PPS results and obtained from H4LS were investigated using the Byar method for rate ratio (RR).

**Results:**

On the day of the PPS, HAI prevalence was 1.47% (95% confidence interval, CI 0.38–3.97), whereas the H4LS incidence rate was 3.53 per 1000 patient-days (PDs, 95% CI 2.99–4.08). Conversion of prevalence rates obtained through the PPS into incidence using the ECDC formula resulted in a rate of 0.86 per 1000 PDs (95% CI 0–2.68). Comparing the two rates, a RR of 0.24 (95% CI 0.03–2.03, p 0.1649) was found.

**Conclusions:**

This study did not find significant differences between HAI incidence estimates obtained from a longitudinal study and through conversion from PPS data. Results of this study support the validity of the ECDC method.

## Introduction

Residents of long-term care facilities (LTCFs) are a population at high risk of developing severe healthcare associated infections (HAIs), as they represent an elder and frail population, often affected by numerous comorbidities and with high healthcare exposure, residing in settings with limited infection prevention and control (IPC) resources [1]. HAIs in LTCF residents are associated with increased risk and length of hospitalization, increased risk of death, and increased healthcare costs [2]. These aspects have implications on the broader health system, not only in terms of the number of patients requiring acute care. LTCFs can act as reservoirs for antimicrobial resistant (AMR) pathogens, and residents transferring across facilities providing different levels of care have the potential of amplifying the spread of AMR pathogens throughout a health system, as seen for *Candida auris* [3]. Further, the treatment of HAIs acquired in LTCFs is a growing indication for antibiotic use in acute-care facilities, which in turn increases antibiotic pressure [4].

Assessing the burden of HAIs requires therefore an integrated approach, including both acute-care settings and LTCFs. The European Centre for Disease Prevention and Control (ECDC) coordinates repeated point prevalence surveys (PPS) of HAIs across European countries in both settings. Based on results of the third Healthcare-Associated Infections and Antimicrobial Use in Long-Term Care Facilities (HALT-3) project, over 4 million HAI episodes among residents of European LTCFs were estimated to have occurred in 2016, amounting to 129,940 residents with at least one HAI on a given day [5].

Incidence estimates obtained from PPS data have been used to assess the burden of HAIs in acute-care hospitals in terms of disability-adjusted life years (DALYs) [6, 7]. Measuring disease burden in DALYs allows a more comprehensive evaluation compared to mortality, as DALYs add years lived with disability (YLD, with a specific disability weight attributed to each health condition) to years of life lost (YLL). Further, calculating DALYs allows to compare disease burdens of very different health conditions, including but not limited to other communicable diseases. Calculating DALYs using an incidence-based approach allows a more consistent measurement of YLL and YLD, and is generally considered more appropriate for acute conditions, such as infectious diseases [8]. However, longitudinal studies of HAI incidence are relatively more resource-intensive compared to PPS, which is particularly true for the LTCF setting. In fact, very few HAI incidence studies conducted in LTCFs have been published to date [9, 10].

**Fig 1:**
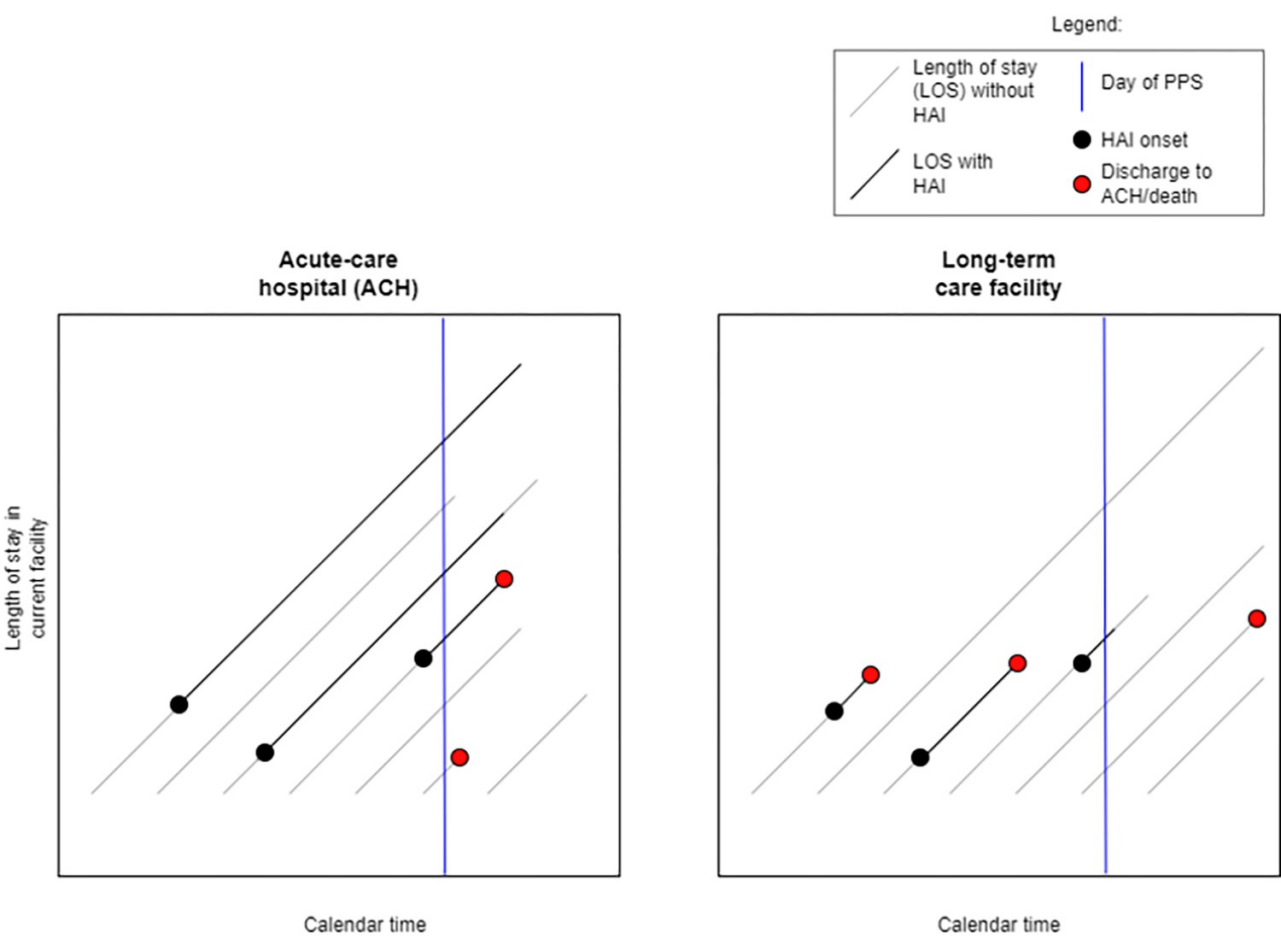
Lexis diagrams illustrating the study hypothesis. Diagonal lines represent the length of stay of each patient. Grey lines represent length of stay (LOS) without infection. Black dots represent the occurrence of a healthcare-associated infection (HAI), and black lines represent the duration of infection. Vertical blue lines represent the day of the point prevalence survey (PPS), in which patients are sampled. The study hypothesis is that compared to acute-care settings, where patients with HAIs are over-represented in PPS due to their longer LOS, residents of long-term care facilities developing HAIs are under-represented, as they may have a greater chance of being transferred to acute-care hospitals (ACH) or of dying (red dots).

For these reasons, DALYs have been calculated by converting HAI prevalence obtained from PPS into incidence, using a modified version of the Rhame and Sudderth formula [11]. This method requires a certain degree of approximation due to the cross-sectional nature of PPS: in particular, residents are assumed to permanently reside in LTCFs, and HAI duration is assessed by multiplying by 2 the median duration from day of onset to the day of the PPS for each specific HAI type [5]. Further, in the assessment of HAIs in acute-care hospitals, selection bias can occur due to cases being over-represented: in fact, patients developing HAIs usually have longer lengths of stays compared to controls, and therefore have an increased probability of being sampled in PPS, leading to an overestimation of HAI prevalence [12, 13]. Our hypothesis was that in LTCFs, the opposite may occur: residents developing HAIs either may have a greater chance of being transferred to acute-care facilities or of dying, and therefore could be under-represented in PPS, leading to an underestimation of HAI prevalence (Fig 1).

To test this hypothesis, we compared results from two studies conducted simultaneously in four LTCFs in Italy: a longitudinal study promoted by the ECDC (HALT4 longitudinal study, H4LS), and a pilot PPS, which applied the same HAI definitions. Our objective was to validate the prevalence to incidence formula proposed by the ECDC in the LTCF setting, to evaluate whether resulting rates would be underestimated in comparison with HAI rates observed in the longitudinal study.

## Material and methods

### Data sources

Data were obtained from LTCFs participating simultaneously in two studies, respectively cross-sectional (pilot PPS) and prospective cohort (H4LS). Both studies were based on the HALT project and applied the same ECDC HAI definitions. Participation was voluntary, and convenience sampling was employed in both studies.

The pilot PPS was conducted between May 15—June 15, 2022. The study was coordinated by the University of Turin in collaboration with the National Health Institute (Istituto Superiore di Sanità, ISS) within a broader project aiming to establish a national HAI surveillance network, financed by the Italian Ministry of Health. The specific objective of the pilot PPS was to establish a surveillance network and to test data collection methods and tools. The study was previously described in detail [14]. Briefly, data were collected in a single day per participating facility at the LTCF, ward, and resident levels. Each facility could pick a date to conduct the PPS within the study window. At the resident level, all active HAIs were recorded, defined as conditions for which signs/symptoms of infection were present on the day of the PPS or for which antibiotic treatment was still ongoing on the day of the PPS.

For the longitudinal study, the H4LS protocol version 1.5 was applied [9]. The study was promoted by the ECDC through the healthcare-associated infections surveillance network (HAI-Net). The national study coordinator for Italy was the Region of Emilia-Romagna. Participating LTCFs were required to prospectively collect data for 12 months, starting January 31^st^, 2022. LTCFs with a median length of stay of 12 months or more were preferred. Within participating facilities, residents present on day 1 of the study were included. Data were collected through institutional, resident, and infection questionnaires. The first two questionnaires were completed at the beginning of the study (including resident clinical characteristics, comorbidities, and risk factors), whereas infection questionnaires were completed for each HAI developed during the follow-up period.

**Fig 2:**
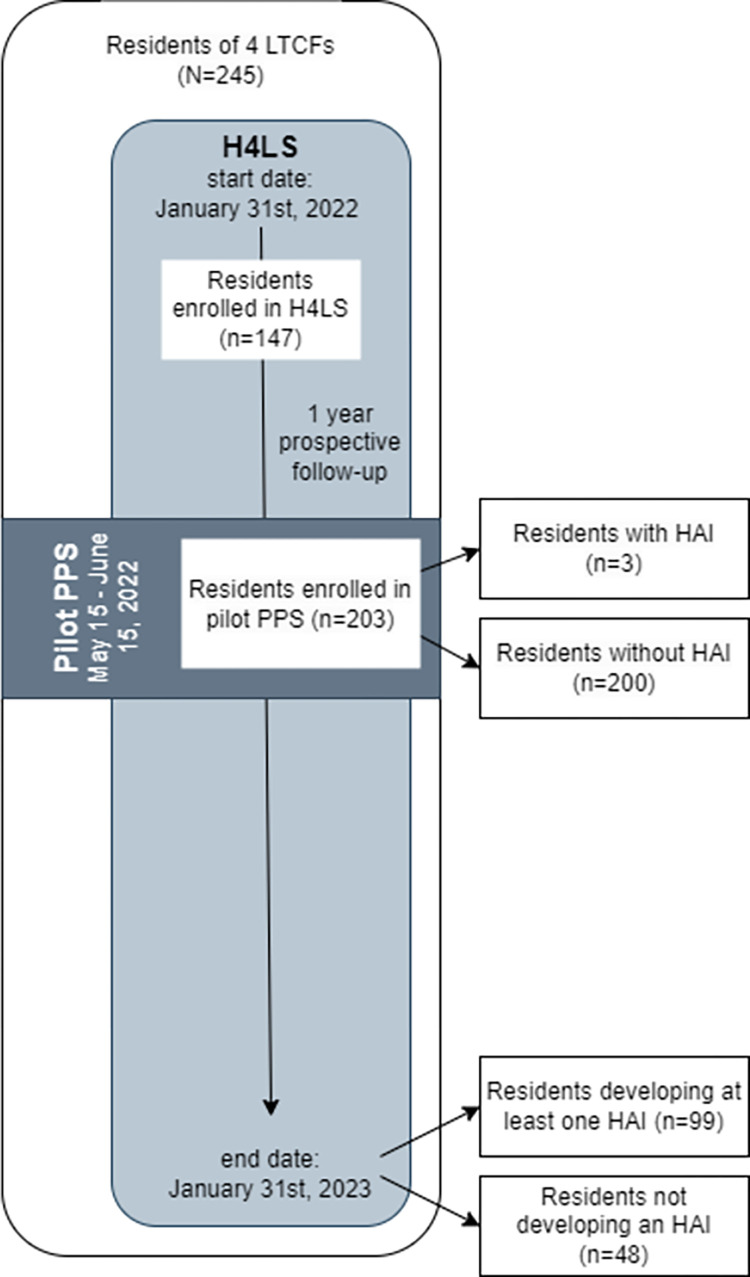
Diagram illustrating the study design. HAI, healthcare-associated infection; H4LS, healthcare-associated infections and antimicrobial use in long-term care facilities longitudinal study; LTCFs, long-term care facilities; PPS, point prevalence survey.

For the purposes of this analysis, data from four LTCFs participating in both studies (PPS and H4LS) were considered. A diagram summarizing the study design is provided in Fig 2.

### Institutional review boards

Both studies were approved by relevant institutions. The pilot PPS was approved by the “Comitato di bioetica d’Ateneo, University of Turin” (protocol n. 0169983, 14 March 2022), “Comitato Etico Interaziendale, Azienda Ospedaliero Universitaria San Luigi Gonzaga, Orbassano” (protocol n. 4611, 21 March 2022) and “Comitato Etico Nazionale per le sperimentazioni degli Enti Pubblici di Ricerca e altri Enti Pubblici a carattere nazionale, Istituto Superiore di Sanità” (protocol n. 0015064, 19 April 2022), in addition to approvals by each appropriate local institutional review board. H4LS received approvals from the “Comitato Etico Interaziendale, Azienda Ospedaliero Universitaria San Luigi Gonzaga, Orbassano” (protocol n. 2720, 18 February 2022) and the “Comitato Etico Area Vasta Emilia Centro della Regione Emilia-Romagna (CE-AVEC)” (protocol n° 929-2021-OSS-AUSLIM, 17 November 2021).

The recruitment period for the pilot PPS coincided with the study period (May 15—June 15, 2022). For the pilot PPS, the need for consent was waived by the ethics committee. Residents were recruited for the longitudinal study from December 1^st^, 2021 to January 31^st^, 2022. Written consent for participation was required from either residents or their caregivers for participation in H4LS.

### Statistical analysis

Descriptive analyses were performed on structure- and patient-level data, using frequencies and medians with interquartile ranges (IQR) for categorical and continuous variables respectively.

Characteristics of residents included in H4LS which developed at least one HAI were compared to those of patients that did not develop infection during the study period, using Fisher and Mann-Whitney U tests when appropriate.

Prevalence was estimated from the pilot PPS in terms of overall prevalence (total number of residents with at least an HAI on the day of the PPS divided by all included residents). Prevalence was converted into incidence per year using an adapted version of the Rhame and Sudderth formula proposed by the ECDC:

$$I=P\times \frac{365}{D\;X\;2}$$ (1)

where I is incidence, P is prevalence, D is the duration of infection (in days), calculated as the median number of days between date of HAI onset and date of the PPS. The formula implies residents are assumed to reside permanently in LTCFs. A correction factor of 95% is applied to account for average occupancy of LTCF beds [5, 11]. The correction factor was calculated by ECDC based on institutional denominator data collected during HALT-3 [5]. Incidence rates were expressed in HAIs per 1000 patient-days (PDs), and respective 95% confidence intervals (CIs) were obtained using Taylor series.

Considering H4LS data, the incidence rate was expressed as the number of HAI episodes over 1000 days of follow-up (PDs), with 95% CI calculated according to the normal approximation to the Poisson distribution, as described by Rosner [15]. Differences between incidence rates calculated from the pilot PPS results and obtained from H4LS were investigated using the Byar method for rate ratio (RR) [16]. Analyses were performed using IBM SPPS v. 28.0.1, setting a two-tailed significance level at α = 0.05.

## Results

### Descriptive statistics

Overall, 15 LTCFs participated in the pilot PPS conducted in May-June 2022. The Italian H4LS sample included 24 LTCFs, and was conducted between January 2022 –March 2023. Four LTCFs (two in the region of Piedmont, and two in the region of Emilia-Romagna) participated in both studies and were therefore included in our analysis. Facility-level descriptive characteristics for each of the LTCFs are provided in Table 1.

**Table 1:** Structure-level characteristics from long-term care facilities (LTCFs) participating in both the pilot point prevalence survey (PPS) and longitudinal study (H4LS) of healthcare-associated infections (HAIs).

	LTCF 1	LTCF 2	LTCF 3	LTCF 4
Facility type	Residential home	Residential home	Mixed LTCF	Mixed LTCF
Ownership	Not-for-profit	Not-for-profit	Private	Public
Size (total number of beds)	60	88	38	59
Number of residents included in pilot PPS (% of total beds)	52 (86.7%)	59 (67%)	38 (100%)	54 (91.5%)
Number of residents included in H4LS (% of total beds)	53 (88.3%)	64 (72.7%)	10 (26.3%)	20 (33.9%)
Study periods:				
H4LS start-end dates; total number of patient days	16/02/22-28/02/23; 15793	01/03/22-28/02/23; 18408	01/03/22-01/03/23; 3616	01/02/22-01/03/22; 7462
		08/06/2022		
Date of pilot PPS	24/05/2022			
			29/07/2022	27/07/2022
Are medical activities in the facility coordinated by a physician?	Yes, internal	Yes, internal	Yes, external	No
Is nursing assistance available 24 hours per day?	Yes	No	No	No
Is personnel trained in infection prevention and control (IPC) working in the facility?	Yes	No	No	No
Does the LTCF have an external or internal IPC committee?	Yes	Yes	Yes	Yes
Is a surveillance programme of HAIs in place in the facility?	Yes	No	Yes	Yes
Are laboratory tests routinely performed to diagnose infections?	Yes	Yes	Yes	Yes

HAI, healthcare-associated infection; H4LS, healthcare-associated infections and antimicrobial use in long-term care facilities longitudinal study; IPC, infection prevention and control; LTCFs, long-term care facilities; PPS, point prevalence survey.

Two of the facilities were classified as residential homes: residents are unable to live independently, and require supervision and assistance for the activities of daily living. The other two facilities were mixed LTCF, providing different types of care within the same facility. The proportion of residents included in the pilot PPS ranged from 67% to 100%, and from 26.3% to 88.3% for H4LS. Reasons for exclusion were mainly due to difficulties in obtaining informed consent from the residents themselves or from their care-givers. IPC resources and practices were uneven, with only one LTCF employing personnel trained in IPC. However, all LTCFs reported having an IPC committee and routinely performing laboratory tests to diagnose infections.

Resident-level characteristics from H4LS are provided in Table 2. Residents developing at least one HAI during follow-up had a higher median Charlson score compared to residents which did not develop HAI, and required hospitalization more frequently (15.2% vs. 4.2%). Hospitalizations among HAI residents also lasted longer. On the other hand, at end of follow-up a higher proportion of patients not developing HAIs were deceased compared to patients developing HAIs, which was the only difference reaching statistical significance among investigated variables (p 0.030).

**Table 2:** Resident-level characteristics and outcomes from the HALT4 longitudinal study (H4LS), stratified according to the occurrence of healthcare-acquired infections (HAIs) during the study period; N = 147.

	Residents with at least one HAI (N = 99)	Residents not developing HAI (N = 48)	All residents included in H4LS
Male gender, n (%)	18 (18.2%)	14 (29.2%)	32 (21.8%)
Age, median (IQR)	88 (83–92)	84.5 (75.8–91.3)	87 (81–92)
Median length of stay prior to study start, median months (IQR)	16 (6–50.5)	24.5 (13–52.3)	20 (8–51)
Charlson score, median (IQR)	2 (1–3)	1 (1–3)	2 (1–3)
Disoriented, n (%)	74 (74.7%)	42 (87.5%)	116 (78.9%)
Using a wheelchair or bedridden, n (%)	79 (79.8%)	39 (81.3%)	117 (79.6%)
Urinary and/or faecal incontinence, n (%)	83 (83.8%)	42 (87.5%)	125 (85%)
Urinary catheter, n (%)	4 (4%)	2 (4.2%)	6 (4.1%)
Vascular catheter, n (%)	2 (2%)	0 (0%)	2 (1.4%)
Previous SARS-CoV-2 infection, n (%)	60 (60.6%)	27 (56.3%)	87 (59.2%)
Residents requiring hospitalization during the study period and reason, n (%)			
Medical	12 (12.1%)	1 (2.1%)	13 (8.8%)
Surgical	1 (1%)	1 (2.1%)	2 (1.4%)
Diagnostic procedures	0 (0%)	0 (0%)	0 (0%)
Unknown	2 (2%)	0 (0%)	2 (1.4%)
Total number of hospitalizations			
1	13 (19.19%)	1 (2.1%)	14 (9.5%)
2	1 (1%)	1 (2.1%)	2 (1.4%)
>3	1 (1%)	0	1 (0.7%)
Length of hospitalization, median days (IQR)	6 (2–12)	3 (1–7)	6 (2–11)
Deceased at end of follow-up, n (%)	19 (19.2%)	18 (37.5%) ^a^	37 (25.2%)

HAI, healthcare-associated infection; H4LS, healthcare-associated infections and antimicrobial use in long-term care facilities longitudinal study; IQR: interquartile range; SARS-CoV-2: severe acute respiratory syndrome Coronavirus 2.

^a^p <0.05 at Chi-squared test.

Characteristics of HAIs are summarized in Table 3. On the day of the pilot PPS, the overall prevalence of HAI was 1.47% (95% CI 0.38–3.97), whereas 67.3% of residents included in H4LS developed at least one HAI during the year of follow-up. Overall, the total number of HAIs recorded during H4LS was 160, resulting in an overall incidence rate of 3.53 per 1000 PDs (95% CI 2.99–4.08). Repeated infections were frequent among patients developing at least one HAI.

**Table 3:** Characteristics of healthcare-associated infections (HAIs) recorded through the pilot point prevalence survey (PPS) and longitudinal study (H4LS).

	Pilot PPS	H4LS
Number of residents with an HAI (% over included residents):		
At least 1 HAI	3 (1.5%)	99 (67.3%)
1 HAI	3 (1.5%)	59 (40.1%)
2 HAI	0	21 (14.3%)
3 HAI	0	12 (8.2%)
4 HAI	0	7 (4.8%)
Overall incidence rate, per 1000 patient-days (PDs)		3.53
Ranking of most frequent HAI types, n (% over all HAIs)		
1°	Skin infections: 1 (33.3%)	COVID-19: 63 (39.4%)
2°	Ear, eye, nose & mouth infections: 1 (33.3%)	Lower respiratory tract infections: 24 (15%)
3°	Other infections: 1 (33.3%)	Urinary tract infections: 23 (14.4%)
Incidence rate of most frequent HAI types, per 1000 PDs		
1° COVID-19		1.39
2° Lower respiratory tract infections		0.53
3° Urinary tract infections		0.51
HAIs with microbiology results, n (% over all HAIs)	0	83 (51.9%)
Most frequently isolated organisms, n (% over HAIs with microbiology results)		
1° SARS-CoV-2	0	63 (75.9%)
2° Escherichia coli	0	5 (6%)
3° Proteus Mirabilis	0	3 (3.6%)
Time from admission to first HAI, median days (interquartile range, IQR)		38 (15–190)
Length of infection, median days (IQR)		7 (5–10)
Infection outcome, n (% of all HAIs)		
Hospitalization		11 (6.9%)
Death (HAI part of causal sequence/contributory cause/sole cause)		1 (0.6%)

COVID-19, coronavirus disease 2019; HAI, healthcare-associated infection; H4LS, healthcare-associated infections and antimicrobial use in long-term care facilities longitudinal study; IQR: interquartile range; PD, patient-day; PPS, point prevalence survey; SARS-CoV-2: severe acute respiratory syndrome Coronavirus 2.

Concerning HAI types, 1 skin infection, 1 ear, eye, nose & mouth infection, and 1 other infection were recorded on the day of the pilot PPS. The most frequent HAI types in H4LS were COVID-19, lower respiratory tract infections, and urinary tract infections. Over half of HAIs in the longitudinal study underwent microbiological testing (for none of which antimicrobial susceptibility testing results were available), and none in the pilot PPS.

According to H4LS results, the median duration of HAI episodes was 7 days (IQR 5–10). The median number of days from HAI onset to the day of the PPS was 9 (range 7–19). In the longitudinal study, 11 HAIs led to residents being hospitalized (6.7% of recorded HAIs) and in one case infection was either part of the causal sequence, contributory cause or sole cause of death (0.6% of HAIs).

### Validation of prevalence to incidence conversion

The calculation of HAI incidence from overall prevalence resulted in a rate of 0.86 per 1000 PDs (95% CI 0–2.68). Comparing this result to HAI incidence obtained from H4LS resulted in a RR of 0.24 (95% CI 0.03–2.03, p 0.1649). Even though this result did not reach statistical significance, the prevalence to incidence conversion method resulted in an underestimation by 1/4 of HAI incidence.

A possible cause for the underestimation of HAI incidence could be due to an overestimation of the duration of HAIs in the cross-sectional survey: in fact, median HAI duration estimated from the pilot PPS was 2 days longer than the duration observed in H4LS. We performed the same calculation using the median HAI duration from H4LS: an incidence rate of 2.22 per 1000 PDs (95% CI 0–5.14) was found, a result much closer to H4LS incidence (RR 0.63, 95% CI 0.17–2.36, p 0.451).

To correct for the overestimation of HAI duration, we propose the following formula for prevalence to incidence conversion:

$$I=P\times \frac{365}{D}$$ (2)

Applying this formula to pilot PPS data (prevalence and HAI duration), we found an incidence rate of 1.73 per 1000 PDs (95% CI 0–4.31), which compared to H4LS incidence resulted in a RR of 0.49 (95% CI 0.11–2.19, p 0.451).

We hypothesized that HAIs with a longer duration have a higher probability of being recorded in PPS compared to shorter HAIs. As we could evaluate the same HAIs through both the longitudinal and cross-sectional survey, we evaluated the probability of HAIs occurring during H4LS of being recorded in the pilot PPS, according to HAI duration (Table 4). None of the HAIs with a duration under 5 days were recorded in the pilot PPS, whereas an increasing probability was found for progressively longer HAIs: 2.08% for HAIs lasting 5–10 days and 2.78% for HAIs lasting > 10 days.

**Table 4:** Probability of healthcare-associated infections (HAIs) occurring during the longitudinal survey (H4LS) of being recorded in the pilot point prevalence survey (PPS), stratifying HAIs according to duration.

HAI duration cathegory	Number of HAIs in H4LS	Number of HAIs recorded in pilot PPS	Probability of HAI being recorded in pilot PPS (%)
< 5 days	28	0	0
5–10 days	96	2	2.08
> 10 days	36	1	2.78
Overall	160	3	1.88

HAI, healthcare-associated infection; H4LS, healthcare-associated infections and antimicrobial use in long-term care facilities longitudinal study; PPS, point prevalence survey.

## Discussion

HAIs in LTCF are a relevant public health and patient safety issue, with potential ramifications throughout the broader health system. This study provided data on HAIs among LTCF residents measured simultaneously through two different study designs, a cross-sectional and a longitudinal study, which applied similar definitions and standardized methods for data collection. Cross-sectional studies allow to estimate prevalence proportions, and by design lack prospective follow-up. Longitudinal designs allow to estimate incidence rates, however require significantly more time and resources [17]. Considering the limited IPC resources of the LTCF setting in particular, this study provided the rare opportunity to validate the methodology proposed by the ECDC for prevalence to incidence conversion, which is an important step in burden estimations [6, 7, 9]. Further, through this study it was possible to formulate hypotheses on the underlying reasons for potential discrepancies between measured incidence and estimates obtained through conversion of prevalence proportions.

Our primary hypothesis for this study was that in the LTCF setting, length-biased sampling seen in acute-care may occur in the opposite direction: residents developing HAIs may be under-represented in PPS, due to a greater chance of being transferred to acute-care facilities or of dying, leading to underestimated HAI prevalence estimates [12]. Comparing HAI incidence obtained from H4LS with the estimate calculated from HAI prevalence from the pilot PPS, no significant difference was found, supporting the validity of the ECDC method. However, the lack of a significant difference could be due to the small number of events seen in the longitudinal study, which is in line with previous reports. An Austrian study with a similar design and which applied definitions based on the ECDC HALT project found an HAI incidence of 2.1 episodes per 1000 patient-days in 2018 (COVID-19 cases were not included) [10].

Regardless of significance, the prevalence to incidence conversion method resulted in an underestimation of one third of HAI incidence, which would have an important impact on burden estimates. A possible explanation could be the overestimation of HAI duration in PPS. As aforementioned, PPS do not allow to gather prospective information. The ECDC method assumes that the time interval between HAI onset and date of the PPS represents 50% of HAI duration. However, as suggested by results of this study, longer HAIs may have an increased probability of being recorded in PPS. Therefore, we propose the simple solution of slightly altering the conversion formula, by only taking into account the length of infection actually measured through the PPS.

When applying the corrected formula, we still found HAI incidence remained underestimated by 50%. Another solution could be applying inverse probability weighting, as proposed for correcting length-biased sampling in PPS of HAIs in acute-care hospitals [12]. Further, Doerken *et al*. have previously suggested that PPS of HAIs in acute-care hospitals should also collect some follow-up data from included patients, namely any incident HAIs and discharge dates [17]. Prospectively collecting data on HAI duration could also improve estimates, and would require relatively less efforts compared to conducting a longitudinal study.

The main limitation of this study was the limited sample size. The small sample size is a concern in the interpretation of the results, affecting generalizability, the lack of precision and reliability and the risk of random variability. Further, participation of LTCFs in both projects was voluntary, therefore we cannot exclude selection bias and make no claim of the representativeness of our results. Another important limitation was the requirement of informed consent for participation in the longitudinal study, which may have introduced additional selection bias.

In conclusion, this study did not find significant differences between HAI incidence estimates obtained from a longitudinal study and through conversion from PPS data. Given the importance of burden estimates in informing policy-makers on rational allocation of healthcare resources, we hope to have contributed to achieving more accurate measurements of HAI incidence in LTCF settings. Further studies applied in a larger sample size could provide more reliable and accurate results.
